# The Association of *VDR/FokI* Gene Polymorphism and Protein Expression With Histopathological Alterations in Patients With Thyroid Colloid Nodule

**DOI:** 10.1155/ancp/6796922

**Published:** 2025-02-18

**Authors:** Mariwan F. Abdalfatah, Abdullah A. Shareef, Lana S. Saleh, Mustafa F. Rajab, Shukur W. Smail, Saman S. Abdulla, Harem Khdir Awla, Rivan H. Ishaac, Kazhal S. Ibrahim, Mazyar J. Ahmed, Fairuz A. Kakasur, Khder Hussein Rasul

**Affiliations:** ^1^Medical Laboratory Science Department, Halabja Technical College, Sulaimani Polytechnic University, Sulaymaniyah, Kurdistan Region, Iraq; ^2^Department of Medical Laboratory, Kurdistan Technical Institute, Sulaymaniyah, Kurdistan Region, Iraq; ^3^Department of Biology, College of Science, Salahaddin University-Erbil, Erbil, Kurdistan Region, Iraq; ^4^Department of Pharmacy, College of Pharmacy, Cihan University-Erbil, Erbil, Kurdistan Region, Iraq; ^5^Department of Oral Diagnosis and Oral Medicine, College of Dentistry, Hawler Medical University, Erbil, Kurdistan Region, Iraq; ^6^Department of Radiological Imaging Technologies, College of Health Technology, Cihan University-Erbil, Erbil, Kurdistan Region, Iraq; ^7^Department of Pathology, Rizgary Teaching Hospital, Ministry of Health, Erbil, Kurdistan Region, Iraq; ^8^Department of Medical Laboratory Sciences, College of Sciences, University of Raparin, Ranya, Kurdistan Region, Iraq; ^9^Kurdistan Higher Council for Medical Specialties, Ministry of Health, Erbil, Kurdistan Region, Iraq; ^10^Medical Analysis Department, Tishk International University, Erbil, Iraq

**Keywords:** colloid nodule, ELISA, fokI, rs2228570, vitamin D polymorphism

## Abstract

**Objective:** Colloid nodules are common and benign thyroid lesions that usually progress slowly and are asymptomatic. It requires follow-up because untreated colloid nodules may develop into malignant tumor. The study aimed to examine the contributions of vitamin D receptor (VDR) expression, VDR/FokI (rs2228570) genotypes, and serum vitamin D level to the susceptibility to colloid nodules.

**Methods:** One hundred forty subjects (80 patients and 60 controls) were enrolled and VDR FokI was determined by PCR in formalin fixed paraffin embedded (FFPE) blocks of the patients and blood of controls. Moreover, VDR protein expression was evaluated by immunohistochemistry using specific VDR monoclonal antibody in the tissue sections of patients and serum vitamin D were measured simultaneously using enzyme-linked immunosorbent assay (ELISA).

**Results:** Sixty-two (77.5%) cases showed strong immunoreactivity score (IRS) of cytoplasmic staining. Strong IRS were significantly observed in samples with larger nodule size (*p* value: 0.0094), multinodules (*p* value: 0.0054), and carriers of CC genotypes (*p* value: 0.0034). TT homozygous genotype revealed significantly (*p* value: 0.029 and odds ratio (OR): 0.11) protective factor for colloid nodules. In addition, nodule size was significantly (*p* value: 0.016) larger among CC carriers. Moreover, vitamin D level and category were nonsignificantly difference between patients and controls.

**Conclusion:** Our results reveal prominent cytoplasmic VDR expression, suggesting a distinct distribution pattern and offering valuable insights into its potential role in colloid nodules. VDR expression increases with increasing size and number of nodules. Regarding FokI genotypes, TT genotype was less likely to develop colloid nodule. These findings contribute to our understanding of cellular characteristics of this condition and may have implications for future research and clinical management.

## 1. Introduction

Thyroid colloid nodules are made up of follicles that are irregularly enlarged and filled with an amount of colloid. Colloid nodule can occur as either single or multiple masses. The nodules vary greatly in size. Some of these nodules can be cystic that identified as cystic colloid nodule that may exhibit characteristics, such as the presence of hemorrhagic or calcified areas [1, 2]. A prolonged decline in TSH stimulation is the most significant contributor to the development of colloid goiter. These have a variety of pathological names, including adenomatous, hyperplastic, and colloid nodules [3]. Physical examination is crucial for the diagnosis of colloid nodules. Only nodules 1 cm in size or larger can be noticed by diligent thyroid palpation if they are located anteriorly [4]. The expression of genes involved in calcium metabolism, cell proliferation, differentiation, apoptosis, and immunity are regulated by the vitamin D receptor (VDR), which is stimulated by vitamin D [5, 6]. Hence, the level of serum vitamin D, tissue VDR expression and *VDR* gene polymorphism contributes to the pathogenesis of many diseases.

The VDR gene located chromosome 12 has many polymorphisms that have been documented to be linked to a variety of physiological and pathological phenotypes in many populations [7]. The most researched gene polymorphisms in the *VDR* gene are Apal (rs7975232), BsmI (rs1544410), Fokl (rs2228570), and Taql (rs731236). The three polymorphisms (ApaI, BsmI, and TaqI) do not lead to any change in VDR protein structure. ApaI and BsmI are intronic, lying in intron 8 [8]. In addition, the polymorphism lies in exon 9 (TaqI) leads to a silent change [9]. However, the FokI variation occurs on exon 2 that leads to a protein with three amino acids shorter [10]. The short 424 amino acid VDR is somewhat more active than the long 427 VDR amino acid, in terms of its transactivation capacity and greater efficiency of binding to vitamin D [11]. The VDR FokI polymorphism (C > T) associated with the autoimmune thyroid diseases [12] and differentiated thyroid carcinoma [13]. Moreover, FokI polymorphism reported to have association with other diseases, including rheumatoid arthritis [14], diabetes mellitus [15], cardiovascular diseases [16], and kidney problems [17]. The epidemiological study conducted in 2021 found that deficiency of vitamin D was connected with thyroid autoantibody positivity [18], and vitamin D supplementation appears to be helpful in the case of thyroid problems [19, 20].

The alteration of VDR expression in the body tissue affects the healthy condition of the tissue. The study revealed decreased expression of VDR by immunohistochemistry in parathyroid adenoma [21]. Methylation of VDR promoter region can alter the expression of VDR [22]. In addition, decreased VDR protein in thyroid tissue associated with hyperparathyroidism [23]. Moreover, low VDR expression measured by IHC staining was associated with aggressive characteristics in different cancers [24]. However, the study related to the role and expression of VDR in benign thyroid nodules are limited. To our knowledge, this was the first attempt to analyze the VDR expression in localized tissue of colloid nodule among Kurdish population. Our aim in this study is to investigate whether specific genotype of *FokI* gene polymorphism (rs2228570) and VDR expression are associated with risk of colloid nodule in Kurdish population and also determining serum vitamin D level in the patients.

## 2. Material and Methods

This is a case–control study conducted in the College of Science–Salahaddin University. A total of 80 patients and 60 healthy controls were enrolled. Only the patients that had been confirmed histopathologically with colloid nodule by experienced pathologists were enrolled. All patients with thyroid cancer or other cancers were excluded. In case of multiple nodules, the largest nodule was counted in. The healthy controls were recruited based on clinically absence of nodule, thyroid dysfunctions, previous thyroid surgery, and family history. Regarding serum vitamin D measurement, blood samples were collected during the same season for all participants to minimize the impact of seasonal variation in sunlight exposure. We excluded individuals whose occupations or hobbies involve significant time spent outdoors, as they have higher and more consistent exposure to sunlight, which could significantly elevate their vitamin D levels. In addition, we excluded individuals with preexisting conditions affecting vitamin D metabolism including liver or kidney disease, eating disorders, and skin diseases. Moreover, we excluded participants with unusually high consumption of vitamin D-rich foods and those taking vitamin D and calcium supplements in the previous month. For the patients, the formalin fixed paraffin embedded (FFPE) blocks and the pathology reports were taken from 2020 to 2022 in pathology laboratories in Erbil city. Nevertheless, the whole blood was taken from the healthy controls for this study. The Ethical Committee of the Department of Biology, College of Science–Salahaddin University (Erbil) approved the research proposal (No: R2345, 25, December 2022) in accordance with Helsinki Declaration.

### 2.1. DNA Amplification and Genotyping

DNA was extracted using 5 mL of blood collected from the controls. The procedure was done according to the manufacturer's protocol (Jena Bioscience, Jena, Germany). DNA extraction from the patients was conducted by taking five sections of 4 µL thickness from FFPE blocks and added to 1.5 mL microcentrifuge tube, then continued using FFPE sample DNA extraction kit (Solarbio, Beijing, China). The NanoDrop spectrophotometer (Thermo Fisher Scientific, Waltham, United States) was used for determination of DNA quantity and purity. For FokI rs2228570, allele specific polymerase chain reaction (AS-PCR) technique was done using three primers previously designed [25], including FokI/F 5′-TGGCCGCCATTGCCTCCG-3′ detecting C allele, FokI/f 5′-TGGCCGCCATTGCCTCCA-3′ detecting T allele, and FokI/C 5′-AGCTGGCCCTGGCACTGA-3′ as a common primer. During PCR, a reaction of 20 µL was added into two 0.2 mL PCR tubes for each sample. One tube contains FokI/F (1 µL) primer and other tube contained FokI/f ((1 µL), then completed to 20 µL by adding 1 µL FokI/C primer, 10 µL Ampliqon master mix, 2 µL DNA sample, and 6 µL nuclease free water for each tube. The amplification was started with initial denaturation (95˚C for 5 min) followed by 35 cycles of denaturation (94˚C for 40 s), annealing (63˚C for 1 min), extension (72˚C for 40 s), and terminated with final extension (72˚C for 5 min). Then, the PCR product was subjected to 2% agarose gel electrophoresis prepared with 1 × TBE buffer and DNA safe stain and run for 30 min (10 min with 45 V, then 20 min with 135 V). The amplified band was visualized using UV trans-illumination (Syngene, Cambridge, UK). The 77 bp amplified band was determined by 50 bp DNA ladder.

### 2.2. Immunohistochemical Staining of VDR

Ultravision detection system anti-polyvalent, HRP kit (Thermo Fisher Scientific), and the specific VDR (D-6) mouse monoclonal antibody (sc-13133; Santa Cruz Biotechnology, Dallas, USA) at 1:100 dilution were used to detect VDR in patients and applied to paraffin-embedded colloid nodule specimen sections according to manufacturer's protocol. Counterstained slides were evaluated by two independent qualified pathologists who were blinded to the patient's clinical characteristics. The expression of VDR were determined in cytoplasm and membrane of the cells by scoring method. The intensity (SI) graded into four categories (0 = no staining, 1 = weak, 2 = moderate, and 3 = strong) and the percentage of stained cells (PP) graded into five classes (None = 0, <10% = 1, 10%–50% = 2, 51%–80% = 3, and >80% = 4). The SI and PP were summed to produce immunoreactivity score (IRS). Score of ≥2 was taken as positive expression (2 = weak, 3–4 = moderate, and 5–7 = strong) [26, 27].

### 2.3. Enzyme-Linked Immunosorbent Assay (ELISA)

5 mL blood samples of each participant were centrifuged to separate serum. Then, specific ELISA kit was used to determine the serum concentrations of 25-hydroxy vitamin D according to the manufacturer's instructions (Monobind, USA). It is considered deficient when the concentration is <20 ng/mL, insufficient 20–29 ng/mL, sufficient 30–100, and potential toxicity >100 ng/mL.

### 2.4. Statistical Analysis

Data distributions were evaluated for normality by the normality test (Kolmogorov–Smirnov test). The difference and comparison between groups was determined using Fisher's exact test, *χ*^2^, *t*-test, ANOVA, odds ratio (OR), and 95% confidence interval (CI). Unpaired *t*-test and Mann–Whitney *U* test were used to compare differences between two unpaired parametric and nonparametric groups, respectively. *p* values < 0.05 were considered statistically significant. The continuous data were expressed as mean ± standard deviation (SD) or median with interquartile range (25%–75%) for data not normally distributed. To demonstrate the stability of allele and genotype frequencies in the Kurdish population, the online Hardy–Weinberg equilibrium (HWE) was employed as a population genetics tool [28].

## 3. Results

In the present study, the patients had median age of 52 years (interquartile range: 39–62) and the median age of the control group was 36 years (interquartile range: 26.5–44.5). The ratio of female to male patient and healthy contributors were 4:1 and 1:1, respectively. The median concentration of vitamin D was 46.10 ng/mL in patients and 42.36 ng/mL in healthy controls. Solitary thyroid nodule to multinodular ratio was 1:3. In addition, other thyroid colloid nodule patient's characteristics which include nodule size, total thyroidectomy (TThy), hemithyroidectomy (HThy), incident malignancy, and IRS are shown in Table 1.

**Figure 1:**
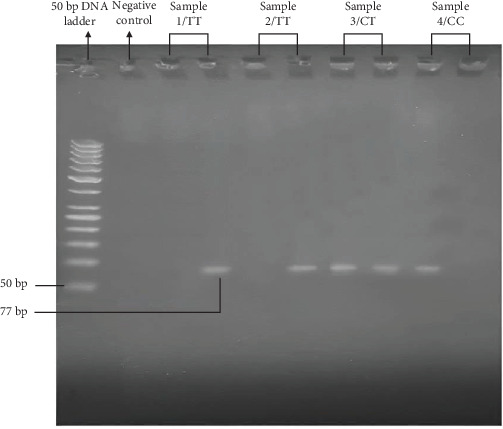
Agarose gel electrophoresis image of amplified PCR products of *FokI* gene polymorphism. Lane 1 is a 50 bp DNA ladder. Lane 2 is a negative control without any sample. Sample 1 and 2 are TT homozygous. Sample 3 is CT heterozygous. Sample 4 is CC homozygous.

In current study, PCR products of three primers showed satisfactory results for DNA extracted from archival paraffin-embedded tissues. Amplified PCR products of FokI gene polymorphism is 77 bp (Figure 1). In addition, the genotype and allele frequencies of *VDR* gene polymorphism FokI (rs2228570) of all participants are demonstrated in Table 2. The genotype frequencies of FokI were not in the HWE (*p*-value < 0.0001) for the patients, but it is in equilibrium (*p*-value = 0.535) for healthy controls. Out of the 140 subjects analyzed for FokI TT homozygous, CT heterozygous, and CC homozygous, the most frequently identified genotype in both patients and healthy individuals is CC homozygous. The CT heterozygous genotype showed nonsignificantly risk factor for colloid goiter. FokI TT homozygous genotype revealed significantly protective factor in colloid goiter patients. Concerning to risk factor or protective factor of genotypes in dominant model, the combined CT + TT genotypes showed slightly higher frequencies in controls (31.66%) than in patients (27.5%), but this difference was not statistically significant (OR = 1.22 and *p*=0.707), indicating that CT + TT does not significantly risk factor for colloid goiter. However, in recessive mode, The TT genotype showed a significantly lower frequency in patients (15%) compared to controls (1.66%), supporting its role as a protective factor with a highly significant *p*-value (OR = 0.09 and *p*=0.0071). Regarding the frequency of alleles, the most frequently identified allele of FokI is C in both groups (patients and controls). Furthermore, no significant differences in the distribution of protective T allele of FokI observed between involved two groups.

**Figure 2:**
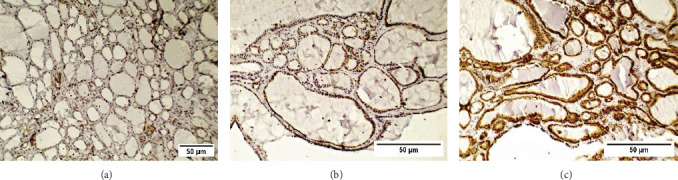
Photomicrographs of immunohistochemichal staining of vitamin D receptor (VDR) expression in colloid nodular goiter. (A–C) Figures show weak, moderate, and strong intensity of staining of thyroid cells, respectively (IHC: A ×100 and B,C ×400; scale bar 50 µm).

The *FokI* gene polymorphism led to different staining intensities of VDR proteins of thyroid cells based on achieved immunohistochemical reactions of VDR expression in colloid nodular goiter (Figure 2). As expected, in all patients, we could not observe cytoplasmic or membranous unstained cells. Additionally, around half of colloid nodular goiter patients had more than 80% cytoplasmic stained cells. While the patients with the highest membranous staining had 10%–50% of stained cells. Also, a highly significant difference was observed between cytoplasmic and membranous stained cells of patients (Table 3). Regarding the staining intensity, all cells of the patients revealed weak or moderate, not strong cytoplasmic and membranous staining intensity. Moreover, only fewer than quarter of patients did not show moderate or weak staining intensities of cytoplasm and membrane of thyroid cells, respectively. Statistical analysis revealed highly significant difference between stained parts of cells of patients regarding staining intensity (Table 4).

The IRS with range of 0–7 to evaluate the staining, 22.5% and 77.5% of patients had 3–4 and 5–7 IRS of cytoplasmic localization, respectively. While, 43.75% and 56.25% of patients had 2 and 3–4 IRS of membranous localization, respectively. The data analysis showed highly significant difference between cytoplasmic and membranous localization of IRS (Table 5).

As shown in Table 6, among the enrolled genotypes and IRS, 3–4 and 5–7 IRS involved most patients with FokI CC homozygous, between these two scores, the 5–7 IRS had higher frequency of patients. In addition, IRS of 5–7 showed significantly increase the size of nodules compared to 3–4 IRS in colloid nodular goiter patients. Moreover, more than half of the included patients were female and multinodular with 5–7 IRS.

Table 7 illustrates the occurrence of demographic and histopathological characteristics of patients according to genotypes. The size of nodules of FokI CC and TT homozygous patients were more and statistically significant compared to CT heterozygous genotypes. Additionally, more than half of the patients were female and multinodular CC homozygous genotype. However, age, gender, and nodularity did not show significant difference between genotypes of patients.

**Figure 3:**
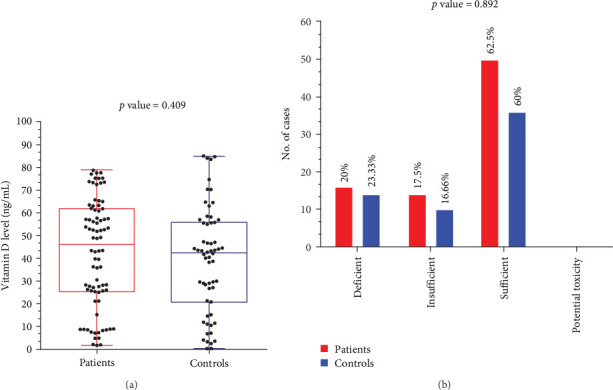
Representing the vitamin D enzyme-linked immunosorbent assay (ELISA) results. (A) Shows the difference in vitamin D concentration between patients and controls (Mann–Whitney test). (B) Shows the difference in vitamin D status between patients and controls (*χ*^2^).

The results obtained by ELISA showed that the median vitamin D concentration of the patients was 46.10 (1.762–78.74) ng/mL and 42.36 (0.075–85.01) ng/mL for control group indicating a nonsignificant difference (Figure 3a). In addition, there was nonsignificant difference (*p* value: 0.892) between the two groups according to vitamin D category. Vitamin D levels were sufficient in most of the participants (patients 65.5% and controls 60%). However, potential vitamin D toxicity was not observed neither in patients nor controls (Figure 3b).

## 4. Discussion

In case of colloid nodule, the deficiency of iodine stimulates thyroid cell hyperplasia that contributes to nodule development which then leads to functional nodule formation that undergoes necrosis and hemorrhage eventually replaced by colloid. Women are more likely than men to have colloid nodules. The nodule could be solitary or develops to multinodules over time [29]. Due to the compression of nearby structures, nodules that become big may cause symptoms [30].

The VDR polymorphism has association with variety of thyroid cancer and the benign thyroid nodules. This study is the first to investigate the involvement of the VDR protein expression and VDR SNP (FokI) in genetic susceptibility to colloid nodular goiter in the Kurdish population. Our results suggest that this SNP has association with thyroid colloid nodule occurrence. The occurrence of thyroid colloid nodule was significantly more prevalent among the people with the TT genotype. This result in line with that in the German population in which variant T (*p*=0.0024) of VDR FokI polymorphism was found to be associated with Graves' disease while variant C (*p*=0.0049) in the Polish population has the association [31]. Concluding that different populations with the same VDR polymorphism show variation in disease occurrence.

Surprisingly, in our study all samples showed positive for VDR staining because colloid nodule is one of the thyroid disorders and it is a benign thyroid lesion. Since the VDR with its ligand (vitamin D) has pleiotropic role such as regulation of inflammation and prevent proliferation, hence, the expression of VDR increases in the abnormal thyroid tissue [32, 33]. The findings of this study are consistent with previous knowledge that cells typically exhibit staining in either the nucleus, cytoplasmic, or membranous regions [34–36]. However, this study demonstrates that the VDR expression is notably higher in the cytoplasm of colloid nodular cells. Thus, this finding provides valuable insights into the cellular characteristics of follicular cells during development of colloid nodules.

In the current study, the higher expression of VDR in the colloid nodule with worse condition of larger and multinodules adds another line of evidence for the role of vitamin D in thyroid diseases. The worse condition of larger nodule and multinodularity require further VDR expression to impede the disease progression that may lead to further nodule formation or developing to malignant tumor [37–39]. Regarding the VDR genotypes, the expression of VDR among CC carriers was greater because they have larger tumor size that require more VDR expression to prevent from bad prognosis. This findings in line with the study by Clinckspoor et al. [40] that showed the high VDR expression in abnormal thyroid tissue such as thyroid cancer and follicular adenoma compare to normal thyroid tissue. The studies reported the relationship of low serum vitamin D level with increased incidence of autoimmune thyroid diseases, thyroid cancer, and benign thyroid nodules [41–44]. This is in contrast with our result in which the patients mostly did not have low vitamin D level. Therefore, the effect of serum vitamin D in patients with colloid nodule needs to be studied further in a larger population that takes into account factors known to affect vitamin D, such as body mass index, race, vitamin D supplementation, and season of measurement.

## 5. Limitations

However, it should be noted that this study is limited by the relatively small sample size and the specific patient population examined. Further research with larger cohorts and diverse patient groups is necessary to validate and expand upon these findings.

## 6. Conclusion

In the Kurdish population, this study offers new evidence for the role of VDR protein expression, VDR SNP (FokI), and blood vitamin D level in the susceptibility to colloid nodule occurrence. Our results demonstrate a significant association between VDR SNP (FokI) and the occurrence of thyroid colloid nodules, with individuals carrying the TT genotype being at lower risk. The findings also indicate that individuals who carry the CC gene variant tend to have larger tumor sizes and need higher level of VDR expression in order to slow down the progression of the disease. This highlights the significance of genetic testing and personalized treatment approaches, for thyroid disorders. Additionally, the study brings attention to the fact that there is a presence of higher VDR protein expression in the cytoplasm of colloid nodular cells. While these findings enhance our understanding of VDR expression patterns in thyroid lesions, their potential diagnostic utility requires further investigation with the inclusion of control tissues. The findings also suggest that vitamin D, through its receptor VDR, may be important in impeding disease progression and preventing the development of malignant tumors, as VDR expression was significantly higher in colloid nodules with worse conditions, such as larger nodules and multinodularity. Future studies may further explore the potential of VDR expression as a prognostic marker for colloid nodule progression and the potential of vitamin D supplementation in preventing and managing thyroid diseases.

## Figures and Tables

**Table 1:** Demographic and histopathological characteristics of the subjects.

Characteristic	Groups	
	Patients *n* = 80	Controls *n* = 60
Age (years)	52 (39–62)	36 (26.5–44.5)
Gender (F/M)	64/16	30/30
Nodularity (solitary/multi)	20/60	—
Nodule size (cm)	3.5 (2.65–4.63)	—
Types of procedure (TThy/HThy)	52/28	—
IRS		
Negative (0–1)	0	—
Positive (≥2)	80	—
Vitamin D (ng/mL)	46.10 (25.4–61.7)	42.36 (20.9–55.8)

*Note:* Median with interquartile range was used for age, nodule size, and vitamin D.

Abbreviations: HThy, hemithyroidectomy; IRS, immunoreactivity score; TThy, total thyroidectomy.

**Table 2:** Genotype and allele frequencies of VDR in the patients with colloid nodular goiter and the healthy participants.

Genotype	Patients *n* = 80	Controls *n* = 60	OR (95% CI)	*p*-Value
CC	58 (72.5%)	41 (68.33%)	1	—
CT	10 (12.5%)	18 (30%)	2.54 (1.06–6.08)	0.0522 ns
TT	12 (15%)	1 (1.66%)	0.11 (0.01–0.94)	0.029^*∗*^
Dominant				
CC	58 (72.5%)	41 (68.33%)	1	—
CT + TT	22 (27.5%)	19 (31.66%)	1.22 (0.58–2.54)	0.707 ns
Recessive				
CC + CT	68 (85%)	59 (98.33)	1	—
TT	12 (15%)	1 (1.66%)	0.09 (0.012–0.761)	0.0071^*∗∗*^
Alleles				
C	126 (78.75%)	100 (83.33%)	1	—
T	34 (21.25%)	20 (16.66%)	0.74 (0.402–1.366)	0.361 ns

*Note:* Fisher's exact test.

Abbreviations: CI, confidence interval; OR, odds ratio; VDR, vitamin D receptor.

*⁣* ^*∗*^ *p* < 0.05. *⁣*^*∗∗*^*p* < 0.01.

**Table 3:** Cytoplasmic and membranous stained cells percentage of colloid nodular goiter patients.

Staining localization	Percentage of stained cells				*p*-Value
	<10% (1)	10%–50% (2)	51%–80% (3)	>80% (4)	
Cytoplasmic	0 (0%)	9 (11.25%)	30 (37.5%)	41 (51.25%)	<0.0001^*∗∗∗∗*^
Membranous	35 (43.75%)	45 (56.25%)	0 (0%)	0 (0%)	

*Note:* Chi-square test.

*⁣* ^*∗∗∗∗*^ *p* < 0.0001.

**Table 4:** Cytoplasmic and membranous staining intensity of colloid nodular goiter patients.

Staining localization	Staining intensity		*p*-Value
	Weak (1)	Moderate (2)	
Cytoplasmic	18 (22.5%)	62 (77.5%)	<0.0001^*∗∗∗∗*^
Membranous	65 (81.25%)	15 (18.75%)	

*Note:* Chi-square test.

*⁣* ^*∗∗∗∗*^ *p* < 0.0001.

**Table 5:** Cytoplasmic and membranous IRS of colloid nodular goiter patients.

IRS	Frequency		*p*-Value
	Cytoplasmic localization	Membranous localization	
Negative (0–1)	0 (0%)	0 (0%)	<0.0001^*∗∗∗∗*^
Weak (2)	0 (0%)	35 (43.75%)	
Moderate (3–4)	18 (22.5%)	45 (56.25%)	
Strong (5–7)	62 (77.5%)	0 (0%)	

*Note:* Chi-square test.

Abbreviation: IRS, immunoreactivity score.

*⁣* ^*∗∗∗∗*^ *p* < 0.0001.

**Table 6:** Genotypic, demographic, and histopathological characteristics according to IRS in colloid nodular goiter patients.

Variables	IRS				*p*-Value
	Negative (0–1)	Weak (2)	Moderate (3–4)	Strong (5–7)	
Genotype					
CC	0 (0%)	0 (0%)	8 (10%)	50 (62.5%)	0.0034^*∗∗*^
CT	0 (0%)	0 (0%)	6 (7.5%)	4 (5%)	
TT	0 (0%)	0 (0%)	4 (5%)	8 (10%)	
Age (years)	—	—	45.5 (43.8–64.8)	52 (36.8–61.8)	0.901^a^ ns
Nodule Size (cm)	—	—	2.41 ± 0.913	3.88 ± 1.58	0.0094^b^*⁣*^*∗∗*^
Gender					
Male	0 (0%)	0 (0%)	3 (3.75%)	13 (16.25%)	0.688 ns
Female	0 (0%)	0 (0%)	15 (18.75%)	49 (61.25%)	
Nodularity					
Solitary	0 (0%)	0 (0%)	0 (0%)	20 (25%)	0.0054^*∗∗*^
Multi	0 (0%)	0 (0%)	18 (22.5%)	42 (52.5%)	

*Note:* Mean ± SD was used for nodule size and median with interquartile range age. Chi-square test.

Abbreviations: IRS, immunoreactivity score; SD, standard deviation.

^a^Mann–Whitney *U* test.

^b^Unpaired *t*-test.

*⁣* ^*∗∗*^ *p* < 0.01.

**Table 7:** Demographic and histopathological characteristics according to genotypes in colloid nodular goiter patients.

Variables	Genotype			*p*-Value
	CC	CT	TT	
Age (year)	52 (27–82)	46 (34–72)	51 (24–62)	0.78^a^ ns
Nodule Size (cm)	4 (0.7–7)	2 (2–3)	3 (0.7–7)	0.016^a^*⁣*^*∗*^
Gender				
Male	8 (10%)	4 (5%)	4 (5%)	0.073 ns
Female	50 (62.5%)	6 (7.5%)	8 (10%)	
Nodularity				
Solitary	16 (20%)	2 (2.5%)	2 (2.5%)	0.675 ns
Multi	42 (52.5%)	8 (10%)	10 (12.5%)	

*Note:* Chi-square test.

^a^Kruskal–Wallis test followed by Dunn's multiple comparisons test.

*⁣* ^*∗*^ *p* < 0.05.

## Data Availability

The datasets used and/or analyzed during the current study are available from the corresponding author on reasonable request.
